# Overlap in synaptic neurological condition susceptibility pathways and the neural pannexin 1 interactome revealed by bioinformatics analyses

**DOI:** 10.1080/19336950.2023.2253102

**Published:** 2023-10-08

**Authors:** Simona D Frederiksen, Leigh E Wicki-Stordeur, Leigh Anne Swayne

**Affiliations:** Division of Medical Sciences, University of Victoria, Victoria, BC, Canada

**Keywords:** PANX1, neurodevelopment, neurodegeneration, synapse, proteomics, gene ontology

## Abstract

Many neurological conditions exhibit synaptic impairments, suggesting mechanistic convergence. Additionally, the pannexin 1 (PANX1) channel and signaling scaffold is linked to several of these neurological conditions and is an emerging regulator of synaptic development and plasticity; however, its synaptic pathogenic contributions are relatively unexplored. To this end, we explored connections between synaptic neurodevelopmental disorder and neurodegenerative disease susceptibility genes discovered by genome-wide association studies (GWASs), and the neural PANX1 interactome (483 proteins) identified from mouse Neuro2a (N2a) cells. To identify shared susceptibility genes, we compared synaptic suggestive GWAS candidate genes amongst autism spectrum disorders, schizophrenia, Parkinson’s disease, and Alzheimer’s disease. To further probe PANX1 signaling pathways at the synapse, we used bioinformatics tools to identify PANX1 interactome signaling pathways and protein–protein interaction clusters. To shed light on synaptic disease mechanisms potentially linking PANX1 and these four neurological conditions, we performed additional cross-analyses between gene ontologies enriched for the PANX1 synaptic and disease-susceptibility gene sets. Finally, to explore the regional specificity of synaptic PANX1-neurological condition connections, we identified brain region-specific elevations of synaptic PANX1 interactome and GWAS candidate gene set transcripts. Our results confirm considerable overlap in risk genes for autism spectrum disorders and schizophrenia and identify potential commonalities in genetic susceptibility for neurodevelopmental disorders and neurodegenerative diseases. Our findings also pinpointed novel putative PANX1 links to synaptic disease-associated pathways, such as regulation of vesicular trafficking and proteostasis, warranting further validation.

## Introduction

Dendritic spines are the site of post-synaptic communication between neurons. Several neurodevelopmental and neurodegenerative conditions, such as autism spectrum disorders (ASD), schizophrenia, Parkinson’s disease, and Alzheimer’s disease, exhibit divergent dendritic spine size, stability, and/or function [1–8]. These alterations often precede obvious clinical symptoms, suggesting they could be involved in disease susceptibility and progression. Despite this, current understanding of the mechanisms affecting dendritic spine dynamics in these conditions is limited.

The pannexin 1 (PANX1) channel and cytoskeleton-regulating protein is emerging as a key regulator of dendritic spines. PANX1, highly expressed at post-synaptic densities [9], oligomerizes to form ion and metabolite channels, and also acts as a channel-independent signaling hub [10]. For example, we discovered protein–protein interactions (PPIs) between PANX1 and key cytoskeleton-regulating proteins involved in dendritic spine formation and stability [11–13], including collapsin response mediator protein 2 (CRMP2) and actin-related protein 3 (ARP3 of the ARP2/3 complex) [14–18]. As follows, PANX1 knock out mice exhibit several synaptic and behavioral abnormalities, such as altered hippocampal long-term potentiation and long-term depression, impaired object recognition, spatial memory and reversal learning, and increased anxiety [19–21]. Consistent with these findings, we discovered that PANX1 limits cortical neuron network size and complexity through inhibition of dendritic spine density and stability [22,23], and similarly, others have shown that PANX1 hinders hippocampal neuron spine maturation [24]. Not surprisingly, PANX1 and/or its protein interaction partners CRMP2 and ARP2/3 are implicated in several neurological conditions associated with dendritic spine abnormalities [25–31], raising the possibility that PANX1 and its interaction partners may contribute to disease risk and/or progression via dendritic spine modulation.

**Figure 1.**
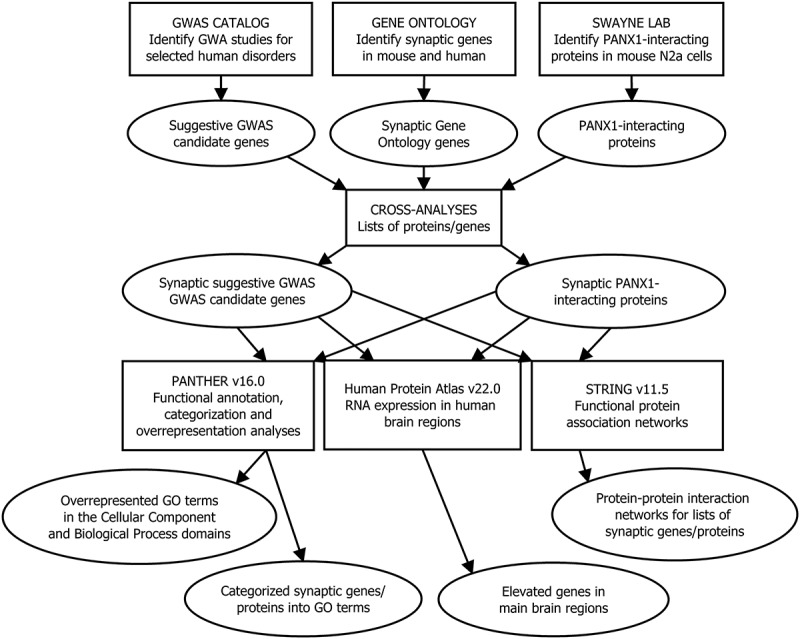
Workflow for the current study from materials and methods to results. In this study, a number of subanalyses were conducted, which explored the neurodevelopmental disorder and neurodegenerative disease susceptibility genes, and the PANX1 interactome in mouse N2a cells overlapping with the gene ontology (GO) synapse. The input data was obtained from three sources: The genome-wide association study (GWAS) catalog (*Homo sapiens*) [41], Swayne lab [our lab] (*Mus musculus*), and the GO knowledge base (*Homo sapiens* and *Mus musculus*) via PANTHER [protein ANalysis THrough Evolutionary Relationships] [42,43], which is a classification system. To enable cross analyses between these datasets, the resources UniProt Retrieve/ID mapping tool, biological DataBase network (bioDBnet) [44], HUGO gene Nomenclature Committee (HGNC) database [45] and mouse genome informatics (MGI) international database [46,47] were used (*e.g.*, to identify human orthologs for mouse UniProtKB IDs). The data were then analyzed using the statistical computing environment R and the following bioinformatics tools/databases: PANTHER [42,48,49], the Human Protein Atlas [50,51] and STRING [Search tool for the Retrieval of interacting genes/proteins] [52–54]. In addition to these subanalyses, the findings were compared to provide a comprehensive overview for each neurodevelopment disorder and neurodegenerative disease in relation to the PANX1 interactome, and overrepresented PANTHER pathways were identified for the PANX1 interactome.

Therefore, here we aimed to identify potential links between synaptic neurological condition susceptibility genes and PANX1 by exploring the extent of overlap in (1) synaptic genes involved in four common neurological conditions associated with broad genetic susceptibility [32–36] and atypical dendritic spines, and (2) these same genes with the neural PANX1 interactome. The neurological conditions we selected, ASD, schizophrenia, Parkinson’s disease, and Alzheimer’s disease, are commonly known and contribute to substantial socioeconomic disease burden [37–40]. Moreover, we performed an in-depth bioinformatics analysis of the neural PANX1 interactome to identify PANX1 interaction partners potentially relevant to dendritic spine dynamics using *in silico* tools (Figure 1). We first cross-referenced findings from Genome-Wide Association Studies (GWASs) for ASD, schizophrenia, Parkinson’s disease, and Alzheimer’s disease, to identify common suggestive risk susceptibility genes. We then performed enrichment analyses of the total neural PANX1 interactome to identify overrepresented biological pathways (*e.g.*, relating to neurological diseases), including implicated PANX1-interacting proteins. We next identified links between existing PPI networks and synaptic PANX1-interacting proteins, the latter obtained by identifying protein hits from our mouse N2a cell PANX1-EGFP interactome annotated to the Gene Ontology (GO) term “synapse”. This was done to gain further insight into the molecular mechanisms that might underlie PANX1 regulation of dendritic spines. To investigate potential links between PANX1 and neurological conditions exhibiting dendritic spine pathology, we compared cellular localizations and biological functions of the synaptic PANX1 interactome with synaptic-enriched susceptibility genes for ASD, schizophrenia, Parkinson’s disease, and Alzheimer’s disease identified by GWASs.

The outcomes of this work provide potential new insights into the role of PANX1 in the central nervous system (CNS) and suggest links between PANX1 and neurological conditions.

## Materials and methods

An overview of the study workflow can be found in Figure 1. For the comparisons and analysis, the R statistical computing environment v4.2.2 was applied. On 2 June 2020, the University of Victoria’s Human Research Ethics Board exempted the study from ethical review as the study: 1) is limited to accessing publicly available data sets and 2) does not involve human participants. Biosafety approval was obtained from the University of Victoria Biosafety committee to undertake the experiments identifying PANX1 interacting proteins. For clarity, it is important to note that according to international conventions (https://www.informatics.jax.org/mgihome/nomen/gene.shtml), all mouse protein abbreviations are fully capitalized. Accordingly, the mouse and human pannexin 1 (also referred to pannexin-1 and pannexin1 in the literature) protein abbreviation is “PANX1.” The gene abbreviation for the mouse pannexin 1 gene is *Panx1,* and the gene abbreviation for the human pannexin 1 gene is *PANX1*. These are the conventions we follow in this manuscript.

### Data inputs

#### Extraction of suggestive GWAS candidate genes involved in neurodevelopmental disorders and neurodegenerative diseases in humans

On 13 November 2022, we searched for the Experimental Factor Ontology (EFO) [55] trait labels “autism spectrum disorder”, “schizophrenia”, “Parkinson disease”, and “Alzheimer disease” separately in the NHGRI-EBI GWAS catalog API [41,56] by means of the gwasrapidd R package [57] and extracted information about the studies (some publications contain multiple GWASs), variants (or single nucleotide polymorphisms; SNPs) and associations (SNP-trait associations). For Parkinson’s disease, 61 studies and 494 associations were available; for Alzheimer’s disease, 117 studies and 1988 associations were available; for schizophrenia, 131 studies, 4961 associations were available; and for ASD, 35 studies and 1275 associations were available. Information about the original publications, including accession ID of the GWAS Catalog study, can be found in Supplemental Table S1.

Using the extracted data, associations without gene information were excluded (gene symbols or entrez IDs; symbols beginning with LOC were kept) and only suggestive GWAS candidate genes reported more than once were included for further analysis. Based on these filtering criteria, a total of 461, 881, 74, and 86 suggestive GWAS candidate genes were identified for ASD, schizophrenia, Parkinson’s disease, and Alzheimer’s disease, respectively. The complete lists of suggestive GWAS candidate genes obtained after filtering were used as inputs for the cross-analyses.

#### Identification of the PANX1 interactome in mouse N2a cells

We previously identified the putative PANX1 interactome from mouse N2a neuroblastoma-derived cells, using methods that were comprehensively described in that work [11,13]. Briefly, proteins co-precipitating with PANX1EGFP [enhanced green fluorescent protein] or EGFP from N2a cells were identified by the UVIC-Genome BC Proteomics Centre using high-performance liquid chromatography-tandem mass spectrometry (LC-MS/MS) followed by analysis with Proteome Discoverer v1.3.0.339 (Thermo Scientific) and Mascot v2.2 [58] [percolator settings: Max delta Cn 0.05, Target false discovery rate (FDR) strict 0.01, Target FDR relaxed 0.05 with validation based on q-value]. The q-value refers to the minimal FDR at which a peptide spectrum match was accepted [59]. The full list of PANX1-interacting proteins can be found in Supplemental Table S2. The first column contains the UniProt mouse accession ID number and abbreviation, and the second column contains the full protein name. This accession number can be queried at uniprot.org for a full entry on the protein, including information such as alternative names, gene name, function, GO annotations, subcellular location, and post-translational modifications. To identify proteins selectively interacting with PANX1, all proteins co-precipitating with anti-GFP antibody from EGFP-expressing cells were removed from the list of PANX1 interactors. This paradigm was repeated three times, and the results were pooled. The complete list of PANX1-interacting proteins identified from those experiments (not previously published in its entirety) was used as input for the cross-analyses.

### Cross-analysis

#### Cross-analysis of the suggestive disorder/disease susceptibility human GWAS candidate genes and mouse neural PANX1 interactome with the GO term “synapse”

To identify known synaptic genes, genes annotated to the “synapse” GO term were extracted for *Homo sapiens* and *Mus musculus* using the PANTHER database v17.0 and the GO Ontology database released on 1 July 2022 [42,48,49,60,61]. Second, the entrez IDs were obtained by converting UniProtKB IDs from the *Homo sapiens* synapse GO term gene list using the UniProt Retrieve/ID mapping tool (https://www.uniprot.org/id-mapping), and finding the human orthologs for the MGI IDs from the *Mus musculus* GO term gene list using the biological DataBase network (bioDBnet; http://biodbnet.abcc.ncifcrf.gov) [44]. When unable to obtain the entrez IDs, we manually looked up the entrez IDs in the HUGO Gene Nomenclature Committee (HGNC) database [45], or the human orthologs in the Mouse Genome Informatics (MGI) international database [46,47]. The gene lists were combined and used as input for the cross-analyses.

To allow for comparison with the GWAS findings, human orthologs in the form of entrez IDs were identified for the mouse UniProtKB IDs forming the PANX1 interactome using bioDBnet [44]. As described above, for the genes we were unable to obtain entrez IDs, we manually looked up the human orthologs in the MGI international database [46].

Next, we conducted cross-analyses between the GO term “synapse” (GO:0045202; identified using the PANTHER database [42,43,49]) and the (i) suggestive GWAS candidate genes involved in the selected human neurodegenerative diseases and neurodevelopmental disorders, and (ii) proteins comprising the mouse PANX1 interactome. This resulted in the identification of synaptic genes/proteins, and subsequently overlaps between the lists of synaptic suggestive GWAS candidate genes for the neurological conditions and synaptic PANX1-interacting proteins were examined.

### Downstream analyses

#### Functional annotation, categorization, and overrepresentation analyses of the synaptic suggestive GWAS candidate genes and PANX1 interactome using the PANTHER database

Bioinformatics analysis of the synaptic suggestive GWAS candidate genes for each neurodevelopmental disorder and neurodegenerative disease (using *Homo sapiens* Entrez gene identifiers), proteins from our PANX1-interacting protein list (using *Mus musculus* UniProtKB unique identifiers), and synaptic proteins from our PANX1-interacting protein list (using *Mus musculus* UniProtKB unique identifiers or *Homo sapiens* Entrez gene identifiers) was carried out using the curated database PANTHER v17.0 [42,43,49]. The genes/proteins were annotated to (i) PANTHER pathways [62] created using the CellDesigner tool, a modeling tool for biochemical networks [63], and/or (ii) GO terms within the Biological Process and Cellular Component domains from the GO knowledge base [60,61]. Right-tailed Fisher’s exact tests were used to identify overrepresented PANTHER pathways and GO terms (present in greater abundance than would be expected). FDR-corrected p-values <0.05 (to account for multiple testing) were considered statistically significant. In addition, only PANTHER pathways and GO terms with at least 10 annotated proteins/genes were presented (to reduce the likelihood of false positives; to allow for comparison, this was not done for Table 5 and corresponding analysis).

**Figure 2.**
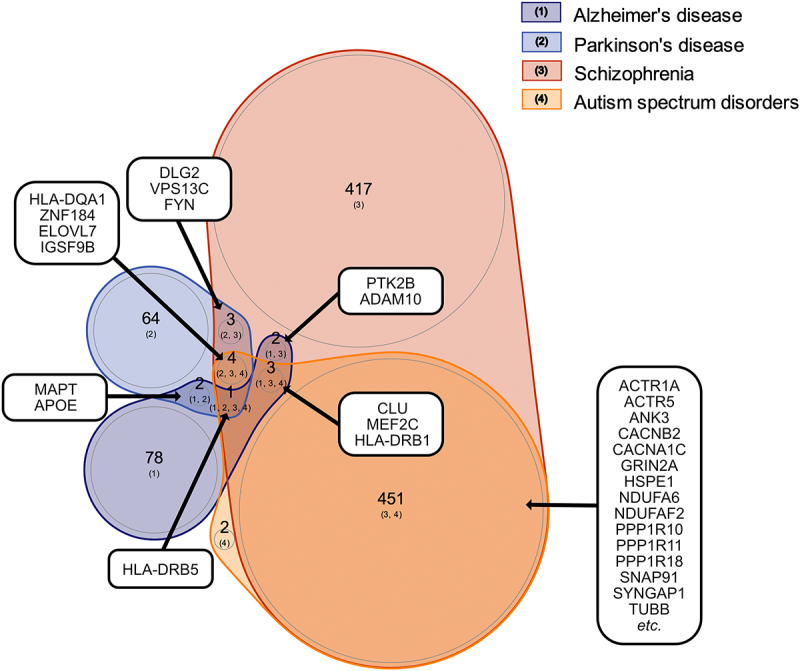
Overlap between genes associated with neurodevelopmental disorders and neurodegenerative diseases in humans based on genome-wide association study (GWAS) findings. Venn diagram displaying the number of suggestive candidate genes the selected neurodevelopmental disorders, autism spectrum disorder (ASD) and schizophrenia, and the selected neurodegenerative diseases, Parkinson’s disease and Alzheimer’s disease, have in common.

#### PPI network for the PANX1 synaptic interactome

The synaptic PPI network, based on interaction evidence [from STRING-defined categories: known interactions (curated databases, and experimentally determined)], was created for *Mus musculus* using the Search Tool for the Retrieval of Interacting Genes/Proteins (STRING) v11.5 [52–54], and the identified PANX1-interacting synaptic proteins were used as input (those proteins overlapping with the *Mus musculus* synapse GO term using the PANTHER v17.0 database and the GO Ontology database released on 1 July 2022 [42,48,49,60,61]). Edges (also termed PPIs) were formed if the interaction score was at least 0.4 (medium confidence, *“estimated likelihood that a given interaction is biologically meaningful, specific and reproducible, given the supporting evidence”* [64]), and the thickness of the edges in the PPI network indicates the strength of data support and dashed line edges reveal PPIs between clusters. First, clusters were identified using the unsupervised Markov Cluster (MCL) algorithm with inflation factor 1.3 (higher inflation factor leads to more clusters but noting that *“MCL is remarkably robust to graph alterations”*) [65–67]. The outcome of this analysis was used to select the final number of clusters in the PPI network, and k-means clustering was carried out. A two-step process was used for quality control purposes (*i.e*., combining bioinformatics approaches and expert knowledge for enhanced confidence in the findings). The potential function of the clusters (formed by at least four proteins) was investigated using the PANTHER database v17.0 [42,48,49] focusing specifically on PANTHER protein classes and GO biological process terms.

#### Synaptic PANX1-interacting proteins and suggestive GWAS candidate genes expressed in specific brain regions in humans

RNA expression in human brain regions was explored using the Human Protein Atlas v22.0 (proteinatlas.org) [50,51], and focusing specifically on genes classified as “regionally elevated.” The findings were compared with the synaptic PANX1 interactome and suggestive GWAS candidate genes for ASD, schizophrenia, Parkinson’s disease, and Alzheimer’s disease. Genes elevated in the following nine brain regions were included in the cross-analysis (UniProtKB IDs were converted to entrez IDs as described above): Cerebral cortex, hippocampal formation, amygdala, thalamus, hypothalamus, midbrain, pons, cerebellum, and medulla oblongata (https://www.proteinatlas.org/humanproteome/brain).

## Results

### Comparison amongst neurological conditions exhibiting impaired synapse structure and/or stability revealed shared synaptic suggestive GWAS candidate genes

**Figure 3.**
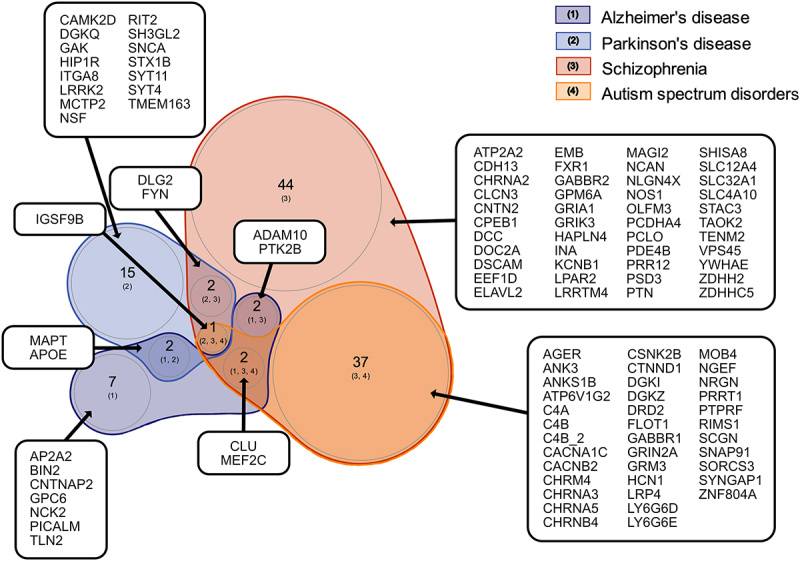
Overlap between genes associated with neurodevelopmental disorders and neurodegenerative diseases in humans based on genome-wide association study (GWAS) findings focusing on those overlapping with the GO “synapse”. Venn diagram visualizing overlap between synaptic suggestive candidate genes associated with the selected neurodevelopmental disorders and neurodegenerative diseases. The synaptic suggestive candidate genes, APOE and MAPT were associated with both Alzheimer’s and Parkinson’s disease, ADAM10 and PTK2B with both Alzheimer’s disease and schizophrenia, DLG2 and FYN with both Parkinson’s disease and schizophrenia, CLU and MEF2C with Alzheimer’s disease, schizophrenia and ASD, and IGSF9B with Parkinson’s disease, schizophrenia and ASD. See table S3 for full lists of genes.

We focused our study on four major neurological conditions exhibiting synapse instability, namely ASD, schizophrenia, Parkinson’s disease, and Alzheimer’s disease. ASD and schizophrenia had more synaptic suggestive GWAS candidate genes in common than any other disease–disorder combination we studied (Figures 2–3; Supplemental Table S3), consistent with other recent findings [68]. In fact, all the synaptic suggestive GWAS candidate genes identified for ASD overlapped with those identified for schizophrenia. These included well-known synaptic genes, such as those encoding for the scaffold-protein ANKG (*ANK3*), the ionotropic glutamate receptor GluN2a (*GRIN2A*), and Ras GTPase activating protein 1 (*SYNGAP1*).

Comparison of the synaptic suggestive GWAS candidate genes across other combinations of diseases and disorders (Table S2) revealed the following overlapping findings: (i) apolipoprotein E (*APOE*) and microtubule associated protein tau (*MAPT*) for Parkinson’s disease and Alzheimer’s disease, (ii) ADAM metallopeptidase domain 10 (*ADAM10*) and protein tyrosine kinase 2 beta (*PTK2B*) for Alzheimer’s disease and schizophrenia, (iii) clusterin (*CLU*) and myocyte enhancer factor 2C (*MEF2C*) for Alzheimer’s disease, schizophrenia, and ASD, (iv) discs large MAGUK scaffold protein 2 (*DLG2*) and FYN proto-oncogene, Src family tyrosine kinase (*FYN*) for Parkinson’s disease and schizophrenia, and (v) immunoglobulin superfamily member 9B (*IGSF9B*) for Parkinson’s disease, schizophrenia & ASD (Figures 2–3; Supplemental Table S3). Based on the STRING database (v11.5), the majority of these genes are directly or indirectly connected either through PPIs (identified from experimental/biochemical data or reported in curated databases) or co-mentioning in PubMed abstracts, indicating involvement in similar biological mechanisms. PANX1 is connected to this STRING network via the proto-oncogene tyrosine-protein kinase (*Src*), which regulates GLUN2A and GLUN2B receptors and synaptic metaplasticity [69] and mediates PANX1-NMDA receptor crosstalk in pathophysiological synaptic plasticity [70,71] through physical interaction [70]. Given that PANX1 is connected to several of these neurological conditions and has been reported to be enriched at post-synaptic membranes, we characterized the neural PANX1 interactome (originally identified by our lab [11,13]) by performing PANTHER pathway and PPI analyses to expand our understanding of its biological roles.

### Bioinformatics analyses of the PANX1 interactome revealed involvement of genes associated with cell structure regulation, proteostasis, neurodegeneration, and synaptic enrichment

In addition to revealing possible biological roles of the PANX1 interactome, we also conducted pathway and PPI analyses to better understand (i) potential implications in disease and (ii) interactions between the PANX1-interacting proteins, based on existing knowledge. PANTHER enrichment analysis of the 483 *Mus musculus* PANX1-interacting proteins (unique UniProt accession numbers) revealed enrichment for six PANTHER pathways, namely the ubiquitin proteasome pathway (P00060, p-value = 3.65E–04), Parkinson’s disease (P00049, p-value = 1.05E–03), integrin signaling pathway (P00034, p-value = 1.61E–03), nicotinic acetylcholine receptor signaling pathway (P00044, p-value = 3.93E–03), inflammation mediated by chemokine and cytokine signaling pathway (P00031, p-value = 1.93E–02) and Huntington disease (P00029, p-value = 1.99E–02). An overview of the PANX1-interacting proteins annotated to the Parkinson’s disease PANTHER pathway can be found in Table 1.

**Table 1.** *Mus musculus* PANX1-interacting proteins annotated to the Parkinson disease PANTHER pathway (P00049), other PANTHER pathways, and PANTHER protein classes.

Gene symbol	Gene description	PANTHER protein class	PANTHER pathway		
			Apoptosis signaling pathway	FGF signaling pathway*	CCKR signaling map**
Hspa1b	Heat shock 70 kDa protein 1B	Hsp70 family chaperone	x		
Hspa1l	Heat shock 70 kDa protein 1-like	Hsp70 family chaperone	x		
Hspa2	Heat shock-related 70 kDa protein 2	Hsp70 family chaperone	x		
Mapk1	Mitogen-activated protein kinase 1	Protein modifying enzyme	x	x	x
Mapk3	Mitogen-activated protein kinase 3	Protein modifying enzyme	x	x	x
Mcm5	DNA replication licensing factor MCM5	DNA metabolism protein	x		
Psma3	Proteasome subunit alpha type-3	Protein modifying enzyme			
Septin1	Septin-1	Cytoskeletal protein			
Septin2	Septin-2	Cytoskeletal protein			
Sfn	14-3-3 protein sigma	Scaffold/adaptor protein		x	
Ywhab	14-3-3 protein beta/alpha	Scaffold/adaptor protein		x	x

Abbreviations: CCKR, cholecystokinin receptor; FGF, fibroblast growth factor. *Involved in nervous system development and maintenance, neuroinflammation and dopaminergic neuron survival rate [72]. **Patients with Parkinson’s disease are more likely to hallucinate if having certain genetic polymorphisms in the CCK gene, and especially when combined with the CCKR, here CCKAR [73].

**Figure 4.**
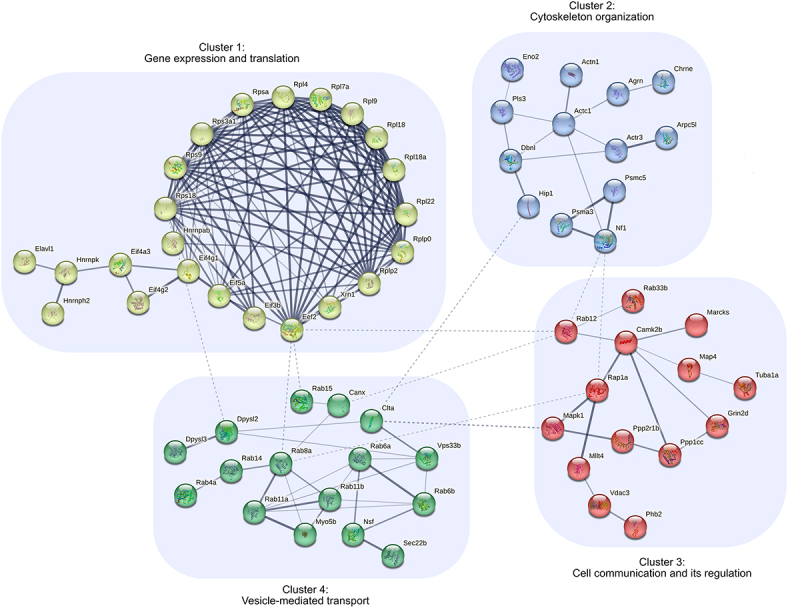
STRING protein–protein interaction (PPI) network to explore the synaptic *Mus musculus* PANX1 interactome and to identify clusters (potential functions presented in Table 2). A total of 66 synaptic PANX1-interacting proteins formed the PPI network (~74% of the synaptic *Mus musculus* PANX1 interactome), based on our chosen methodology. The thickness of the edges in the PPI network indicates the strength of data support and dashed line edges reveal PPIs between clusters.

As PANX1 is involved in a wide range of synaptic functions, we decided to identify synaptic PANX1-interacting proteins (~18% of the neural PANX1 interactome) via cross-analysis with the *Mus musculus* cellular component GO term “synapse.” Using the 89 identified synaptic PANX1-interacting proteins as input, we created a STRING PPI network formed by 66 synaptic PANX1-interacting proteins (Figure 4) and identified 4 clusters involved in gene expression and translation, cytoskeleton organization, vesicle-mediated transport, and cell communication and its regulation, respectively (Table 2). When conducting the cross-analysis with both the *Mus musculus* and *Homo sapiens* synapse GO term (combined), 92 synaptic PANX1-interacting proteins were identified (see Table 3 for an overview of these proteins).

**Table 2.** Synaptic *Mus musculus* PANX1-interacting proteins forming the STRING protein–protein interaction network clusters annotated to selected PANTHER protein classes and gene ontology (GO) biological process (BP) terms.

PANTHER protein class /GO BP term	% of proteins			
	Cluster 1	Cluster 2	Cluster 3	Cluster 4
	*n* = 23	*n* = 13	*n* = 14	*n* = 16
Anatomical structure development (GO:0048856)	26.1	53.8	71.4	37.5
Nervous system development (GO:0007399)	8.7	30.8	50.0	31.3
Gene expression (GO:0010467)	87.0	0.0	7.1	0.0
Translational protein (PC00263)	78.3	0.0	0.0	0.0
Protein transport (GO:0015031)	8.7	7.7	28.6	81.3
Cytoskeleton organization (GO:0007010)	0.0	61.5	21.4	25.0
Cytoskeletal protein (PC00085)	0.0	53.8	14.3	6.3
Cell communication (GO:0007154)	4.3	38.5	64.3	25.0
Regulation of cell communication (GO:0010646)	13.0	23.1	57.1	18.8
Regulation of signaling (GO:0023051)	13.0	23.1	57.1	25.0
Vesicle-mediated transport (GO:0016192)	0.0	15.4	28.6	93.8

**Table 3.** Overview of *Mus musculus* synaptic PANX1-interacting proteins.

UniProt ID	Gene Name	Description
P62196	Psmc5	26S protease regulatory subunit 8
P62270	Rps18	40S ribosomal protein S18
P97351	Rps3a	40S ribosomal protein S3a
Q6ZWN5	Rps9	40S ribosomal protein S9
P97789	Xrn1	5”−3” exoribonuclease 1
P14869	Rplp0	60S acidic ribosomal protein P0
P99027	Rplp2	60S acidic ribosomal protein P2
P35980	Rpl18	60S ribosomal protein L18
P62717	Rpl18a	60S ribosomal protein L18a
P67984	Rpl22	60S ribosomal protein L22
Q9D8E6	Rpl4	60S ribosomal protein L4
P12970	Rpl7a	60S ribosomal protein L7a
P51410	Rpl9	60S ribosomal protein L9
P20782	Chrne	Acetylcholine receptor subunit epsilon
P68033	Actc1	Actin, alpha cardiac muscle 1
Q9D898	Arpc5l	Actin-related protein 2/3 complex subunit 5-like protein
Q99JY9	Actr3	Actin-related protein 3
Q9CQW2	Arl8b	ADP-ribosylation factor-like protein 8B
Q9QZQ1	Mllt4	Afadin
A2ASQ1	Agrn	Agrin
Q7TPR4	Actn1	Alpha-actinin-1
Q91ZU6	Dst	Bullous pemphigoid antigen 1
P28652	Camk2b	Calcium/calmodulin-dependent protein kinase type II subunit beta
P35564	Canx	Calnexin
O08585	Clta	Clathrin light chain A
Q8BZN6	Dock10	Dedicator of cytokinesis protein 10
O08553	Dpysl2	Dihydropyrimidinase-related protein 2
Q62188	Dpysl3	Dihydropyrimidinase-related protein 3
Q62418	Dbnl	Drebrin-like protein
Q91V17	Znrf1	E3 ubiquitin-protein ligase ZNRF1
P70372	Elavl1	ELAV-like protein 1
P58252	Eef2	Elongation factor 2
Q91VC3	Eif4a3	Eukaryotic initiation factor 4A-III
Q8JZQ9	Eif3b	Eukaryotic translation initiation factor 3 subunit B
Q6NZJ6	Eif4g1	Eukaryotic translation initiation factor 4 gamma 1
Q62448	Eif4g2	Eukaryotic translation initiation factor 4 gamma 2
Q9CS72	Filip1	Filamin-A-interacting protein 1
P17183	Eno2	Gamma-enolase
Q03391	Grin2d	Glutamate [NMDA] receptor subunit epsilon-4
P21278	Gna11	Guanine nucleotide-binding protein subunit alpha-11
Q99020	Hnrnpab	Heterogeneous nuclear ribonucleoprotein A/B
P70333	Hnrnph2	Heterogeneous nuclear ribonucleoprotein H2
P61979	Hnrnpk	Heterogeneous nuclear ribonucleoprotein K
Q8VD75	Hip1	Huntingtin-interacting protein 1
Q60960	Kpna1	Importin subunit alpha-1
O35711	Ppfibp2	Liprin-beta-2
P60755	Mdga2	MAM domain-containing glycosylphosphatidylinositol anchor protein 2
P27546	Map4	Microtubule-associated protein 4
P63085	Mapk1	Mitogen-activated protein kinase 1
P53986	Slc16a1	Monocarboxylate transporter 1
P57787	Slc16a3	Monocarboxylate transporter 4
P21271	Myo5b	Myosin-Vb
P26645	Marcks	Myristoylated alanine-rich C-kinase substrate
Q04690	Nf1	Neurofibromin
P26883	Fkbp1a	Peptidyl-prolyl cis-trans isomerase FKBP1A
Q99K51	Pls3	Plastin-3
O35129	Phb2	Prohibitin-2
O70435	Psma3	Proteasome subunit alpha type-3
A2A690	Tanc2	Protein TANC2
P62492	Rab11a	Ras-related protein Rab-11A
P46638	Rab11b	Ras-related protein Rab-11B
P35283	Rab12	Ras-related protein Rab-12
Q91V41	Rab14	Ras-related protein Rab-14
Q8K386	Rab15	Ras-related protein Rab-15
O35963	Rab33b	Ras-related protein Rab-33B
Q6PHN9	Rab35	Ras-related protein Rab-35
Q9CZT8	Rab3b	Ras-related protein Rab-3B
P62823	Rab3c	Ras-related protein Rab-3C
P35276	Rab3d	Ras-related protein Rab-3D
P56371	Rab4a	Ras-related protein Rab-4A
P35279	Rab6a	Ras-related protein Rab-6A
P61294	Rab6b	Ras-related protein Rab-6B
P55258	Rab8a	Ras-related protein Rab-8A
P62835	Rap1a	Ras-related protein Rap-1A
Q9ES97	Rtn3	Reticulon-3
P18654	Rps6ka3	Ribosomal protein S6 kinase alpha-3
P42209	Sept1	Septin-1
P42208	Sept2	Septin-2
Q7TNP2	Ppp2r1b	Serine/threonine-protein phosphatase 2A 65 kDa regulatory subunit A beta isoform
P63087	Ppp1cc	Serine/threonine-protein phosphatase PP1-gamma catalytic subunit
Q91Z67	Srgap2	SLIT-ROBO Rho GTPase-activating protein 2
O35316	Slc6a6	Sodium- and chloride-dependent taurine transporter
Q8BRU6	Slc18a2	Synaptic vesicular amine transporter
O08992	Sdcbp	Syntenin-1
P83510	Tnik	Traf2 and NCK-interacting protein kinase
P68369	Tuba1a	Tubulin alpha-1A chain
A2AAE1	Kiaa1109	Uncharacterized protein KIAA1109
P59016	Vps33b	Vacuolar protein sorting-associated protein 33B
P46460	Nsf	Vesicle-fusing ATPase
O08547	Sec22b	Vesicle-trafficking protein SEC22b
Q60931	Vdac3	Voltage-dependent anion-selective channel protein 3
Q9Z1G4	Atp6v0a1	V-type proton ATPase 116 kDa subunit a isoform 1

### Cross-analyses revealed overlap between the full neural and synapse-specific PANX1 interactome and neurological disorder/disease susceptibility genes

To explore connections between PANX1 and the four disorders/diseases of interest, we compared the entire PANX1 interactome with the suggestive candidate genes for each neurological condition (Figure 2). Overall, the two neurodevelopmental disorder susceptibility gene sets exhibited a relatively larger degree of overlap with the PANX1 interactome than did the neurodegenerative disease susceptibility gene sets. Twenty schizophrenia susceptibility genes overlapped with the PANX1 interactome, which was the largest number in common, while ASD had 12 genes in common with the PANX1 interactome (see Table 4 for an overview). Several of these genes are involved in protein folding (GO:0006457; *Hspa1b, Hspa1l, Hspd1, Hspe1, and St13*) or regulation of translation (GO:0006417; *Cnot1*, *Ddx39b, Etf1, Lsm1, and Mapk3*).

**Table 4.** Genes encoding for PANX1-interacting proteins that have also been identified as suggestive GWAS candidate genes (Figure 2).

Gene symbol	Gene description
**Parkinson’s disease**	
Nsf	Vesicle-fusing ATPase
**Alzheimer’s disease**	
Usp6nl	USP6 N-terminal-like protein
**Schizophrenia**	
Acads*	Short-chain specific acyl-CoA dehydrogenase, mitochondrial
Actr1a*	Alpha-centractin
Agpat1*	1-acyl-sn-glycerol-3-phosphate acyltransferase alpha
Ddx39b*	Spliceosome RNA helicase Ddx39b
Cnot1*	CCR4-NOT transcription complex subunit 1
Csrp2	Cysteine and glycine-rich protein 2
Etf1	Eukaryotic peptide chain release factor subunit 1
Gmip	GEM-interacting protein
Gna12	Guanine nucleotide-binding protein subunit alpha-12
Gnl1*	Guanine nucleotide-binding protein-like 1
Gulo	L-gulonolactone oxidase
Hspa1b*	Heat shock 70 kDa protein 1B
Hspa1l*	Heat shock 70 kDa protein 1-like
Hspd1*	60 kDa heat shock protein, mitochondrial
Hspe1*	10 kDa heat shock protein, mitochondrial
Lsm1	U6 snRNA-associated Sm-like protein LSm1
Mapk3	Mitogen-activated protein kinase 3
Plcl1*	Inactive phospholipase C-like protein 1
Psmd6	26S proteasome non-ATPase regulatory subunit 6
St13*	Hsc70-interacting protein

Note: *Suggestive GWAS candidate genes associated with autism spectrum disorder that overlapped with the PANX1 interactome (complete overlap with the suggestive GWAS candidate genes associated with schizophrenia).

Since synaptic dysfunction has been associated with neurological conditions such as Parkinson’s disease [74], to identify synaptic-specific associations, we compared the (*Mus musculus* and *Homo sapiens* synapse GO term-defined) synaptic PANX1 interactome with (*Mus musculus* and *Homo sapiens* synapse GO term-defined) synaptic suggestive GWAS candidate genes (Figure 3 and Table S2). Based on our methodological workflow (*e.g.*, restricted to genes reported more than once in the GWAS catalog and genes annotated to the *Mus musculus* and *Homo sapiens* synapse GO term), we identified the gene coding for N-ethylmaleimide sensitive factor, vesicle-fusing ATPase (NSF) as both a suggestive candidate gene for Parkinson’s disease and a PANX1-interacting protein. It is important to note that the GO definition of “synapse” does not include several important synaptic proteins, as further elaborated in the discussion.

Therefore, to circumvent the limitations of the restricted GO definition of “synapse” genes in our exploration of PANX1-related disease connections of a synaptic nature, we made a broader comparison between GO terms enriched within the synaptic PANX1 interactome and GO terms enriched for the four neurological conditions (based on synaptic disorder/disease susceptibility genes; Table 5). We included terms associated with the GO domains “cellular component” and “biological process” (choosing domains fitting the aim of this study), as these two relatively broader GO domains could help provide insight into common pathophysiological mechanisms and potential PANX1 involvement. The “cellular component” domain was selected to explore localization to structures associated with synapses, and/or structures influencing synapses and their development or stability (*e.g.,* “somatodendritic tree” should have some bearing on dendritic spines, “cytoplasmic vesicle” would be associated with transport processes required to bring cargo to nascent spines and so on). The following GO terms were enriched for the synaptic PANX1 interactome and each of the neurological conditions: Asymmetric synapse (GO:0032279), glutamatergic synapse (GO:0098978), neuron to neuron synapse (GO:0098984), postsynapse (GO:0098794), postsynaptic density (GO:0014069), and postsynaptic specialization (GO:0099572; Table 5).

**Table 5.** Enrichment analysis (GO terms within the “cellular component” and “biological process” GO domains) for the synaptic PANX1 interactome showing GO terms also found for the investigated neurological conditions (as indicated with X).

Enriched GO term*	PANX1		Autism spectrum disorder	Schizophrenia	Parkinson’s disease	Alzheimer’s disease
	FE	P-value**				
**Cellular component GO domain▲**						
Asymmetric synapse (GO:0032279)	7.07	5.41E–06	**X**	**X**	**X**	**X**
Endocytic vesicle (GO:0030139)	5.25	1.11E–03			**X**	**X**
Exocytic vesicle (GO:0070382)	7.82	5.22E–05	**X**	**X**	**X**	
Glutamatergic synapse (GO:0098978)	8.06	2.12E–07	**X**	**X**	**X**	**X**
Neuron to neuron synapse (GO:0098984)	6.58	1.07E–05	**X**	**X**	**X**	**X**
Postsynapse (GO:0098794)	7.50	9.44E–13	**X**	**X**	**X**	**X**
Postsynaptic density (GO:0014069)	7.20	4.62E–06	**X**	**X**	**X**	**X**
Postsynaptic specialization (GO:0099572)	7.29	1.54E–06	**X**	**X**	**X**	**X**
Presynapse (GO:0098793)	5.14	2.23E–05	**X**	**X**	**X**	
Transport vesicle (GO:0030133)	5.05	2.84E–04	**X**	**X**	**X**	
**Biological process GO domain▼**						
Cell junction assembly (GO:0034329)	6.54	5.93E–03		**X**		**X**
Cell junction organization (GO:0034330)	4.35	1.77E–02	**X**	**X**	**X**	**X**
Establishment of localization (GO:0051234)	2.26	4.96E–05	**X**	**X**	**X**	
Regulation of vesicle-mediated transport (GO:0060627)	4.25	1.23E–02	**X**	**X**	**X**	**X**
Transport (GO:0006810)	2.30	2.75E–05	**X**	**X**	**X**	
Vesicle-mediated transport (GO:0016192)	3.27	5.64E–04		**X**	**X**	

Abbreviations: FE, Fold enrichment (actual number of Panx1-interacting proteins over the expected number of proteins); GO, gene ontology. *Search for the GO term definition here: http://amigo.geneontology.org/amigo/search/ontology **FDR-adjusted p-value (*p* < 0.05). ^▲^Inclusion criteria: GO terms with 10 or more annotated genes/proteins AND with a fold enrichment of at least 5. ^▼^Inclusion criteria: GO terms with 10 or more annotated genes/proteins AND with a fold enrichment of at least 2.

Focusing on the GO “biological process”, two GO terms overlapped (*i.e.*, cell junction organization (GO:0034330) and regulation of vesicle-mediated transport (GO:0060627)) when comparing the synaptic PANX1 interactome and the neurological conditions (Table 5).

### Specific human brain regions associated with ASD, schizophrenia, Parkinson’s disease, and Alzheimer’s disease

Since the neurological conditions we investigated are well known to exhibit spatiotemporal specificity in terms of progression in affected brain regions (for example, Parkinson’s disease initially presents with striatal dysfunction), we investigated regional specificity in elevated transcript expression for the synaptic PANX1 interactome and synaptic GWAS candidate genes by means of cross-analysis. Note that a gene was considered as “expressed” in a given brain region when the normalized transcript per million (nTPM; i.e. expression value) was above 1. Using the Human Protein Atlas v22.0 (proteinatlas.org) [50,51], we found three of the synaptic PANX1-interactors exhibited regionally elevated transcript expression: RPL9 in cerebral cortex, SLC18A2 in pons, midbrain, and hypothalamus, and TANC2 in cerebral cortex, hippocampal formation, and amygdala (Table 6; based on dataset on summarized expression in main brain regions not the specific expression in the more than 200 regions, areas, and subfields, separately). Regionally elevated expression of synaptic neurological disorder/disease susceptibility genes was also found, with the highest number of genes noted for schizophrenia (16) and ASD (11; Table 6). Most of the disorder/disease-associated genes were elevated in the cerebral cortex, cerebellum, hippocampal formation, and/or amygdala (Table 6).

**Table 6.** Overview of the overlap between the elevated genes in specific brain regions (Human Protein Atlas) and the synaptic PANX1 interactome/suggestive neurological disorder/disease susceptibility genes.

Elevated gene	Brain region							
	Cerebral cortex	Cerebellum	Hippocampal formation	Amygdala	Pons	Midbrain	Hypothalamus	Thalamus
CHRM4	SCZ, ASD							
CHRNA2								SCZ
DGKZ	SCZ, ASD		SCZ, ASD	SCZ, ASD				
GRIN2A	SCZ, ASD							
HCN1	SCZ, ASD							
IGSF9B		PD, SCZ, ASD						
KCNB1	SCZ							
MCTP2	PD							
MEF2C	AD, SCZ, ASD		AD, SCZ, ASD	AD, SCZ, ASD				
NRGN	SCZ, ASD							
PCLO		SCZ						
PRRT1	SCZ, ASD		SCZ, ASD	SCZ, ASD				
RIMS1		SCZ, ASD						
RPL9	PANX1							
SCGN		SCZ, ASD	SCZ, ASD				SCZ, ASD	
SHISA8		SCZ						
SLC18A2					PANX1	PANX1	PANX1	
SLC4A10	SCZ							
STX1B	PD							
SYNGAP1	SCZ, ASD		SCZ, ASD	SCZ, ASD				
TANC2	PANX1		PANX1	PANX1				

Abbreviations: AD, Alzheimer’s disease; ASD, autism spectrum disorders; PD, Parkinson’s disease; SCZ, schizophrenia.

## Discussion

The goal of this study was to identify common synaptic genes and molecular pathways amongst neurological conditions and PANX1 using bioinformatics approaches. Using large-scale bioinformatics approaches has many advantages including but not limited to the: (i) ability to efficiently analyze a large amount of data, (ii) approaching analysis beyond tradition (*e.g.,* data integration to get new insights), and (iii) possibility of making novel discoveries. Our study revealed multiple potential links between the PANX1 interactome and the investigated neurological conditions that now warrant validation. Overall, the two investigated neurodevelopmental disorders exhibited the largest overlap in synaptic susceptibility genes and were more abundantly represented in the PANX1 interactome. Our results suggest that the molecular mechanisms underlying synaptic dysfunction in neurodevelopmental disorders may be more closely linked to one another and to PANX1 than is the case for neurodegenerative diseases, although it should be noted that this larger overlap also may be influenced by the size of these gene sets compared with those extracted for the investigated neurogenerative diseases. While the research on PANX1 involvement in neurological conditions has primarily focused on neurodegenerative diseases, it is perhaps not surprising that the PANX1 interactome is linked to neurodevelopmental disorder molecular players, given expanding evidence for overlap between molecular mechanisms underlying neurodevelopmental disorders and neurodegenerative diseases, particularly at the synapse and the role for PANX1 in regulation of synapse development and plasticity [19–24,75]. There is mounting evidence that synaptic dysfunction associated with neurodegenerative diseases can be detected at early pre-symptomatic stages, and there are growing links in neurodevelopmental and neurodegenerative gene expression changes and genetic risk susceptibilities [76–86].

### Further evidence for common genetic elements of ASD and schizophrenia

Notably, several of the susceptibility genes common to ASD and schizophrenia in our analysis have previously been linked to both disorders [87,88] and play key roles at the synapse (reviewed in [1,3,4]). For example, *ANK3* (ANKG protein), *GRIN2A* (GluN2A), and *SYNGAP1* have established synaptic roles [89–92] and connections to ASD, schizophrenia, and other co-morbid neurodevelopmental disorders like epilepsy [93–98]. Although GluN2A was not identified in our PANX1 interactome, we identified the closely related protein GluN2D (*GRIN2D*). PANX1 has been previously reported to form a complex with GluN1 (obligatory NMDA receptor subunit) and Src [70] in juvenile rat brain hippocampal slices. For our interaction screen, we used the N2a mouse cell line derived from a neuroblastoma (neural crest in origin) that is widely used to study cell biological aspects of neuronal differentiation [99]. The relatively immature state of the undifferentiated N2a cell model and the high stringency of our immunoprecipitation conditions (RIPA buffer which contains harsh detergents and preserves only robust interactions) could partially account for the lack of identification of GluN1 as a PANX1 interactor. It is surprising that we would detect GluN2D and not GluN1, but perhaps GluN2D plays an early non-synaptic, non-receptor role that has yet to be identified; this may be consistent with its decrease in levels with increasing age/development [100]. Conversely, the lipid raft scaffolding protein, flotillin, was common to ASD and schizophrenia but has not been extensively studied in either neurodevelopmental disorder. Given lipid raft/cholesterol dysfunction has been implicated in the monogenic common ASD Fragile X syndrome and has been linked to glutamatergic synapse formation [101]; further exploration of the role of flotillin in synaptic dysfunction in ASD and schizophrenia could be informative. Further analysis is now warranted to explore the shared synaptic genetic susceptibility between ASD and schizophrenia, including individual variability in risk, onset, severity, and progression. Some studies have looked into this shared genetic basis. For instance, significant genetic correlations have been identified between ASD and schizophrenia (but not between Alzheimer’s disease and Parkinson’s disease) [68], similar to what we observed in this study. Even though ASD and schizophrenia share genetic risks, they seem to have distinct developmental profiles [102]; multi-omics studies could help tease out the underpinnings.

The established synaptic roles of these neurological disorder/disease susceptibility genes and our recent discovery of PANX1 regulation of dendritic spine stability prompted our synaptic PANX1 interactome STRING analysis, which identified clusters in gene expression and translation, cytoskeleton organization, vesicle-mediated transport, and cell communication and its regulation (Table 2). Dysregulation in these same cellular processes are observed in ASD [103,104], schizophrenia [105,106], Parkinson’s disease [107,108], and Alzheimer’s disease [109,110], and PANX1 has been linked to these conditions (reviewed in [75,111]). Of particular note, there are >40 non-coding *PANX1* variants in the VariCarta ASD database (https://varicarta.msl.ubc.ca/index [112]), and there are several ASD-linked *PANX1* SNPs associated with brain-specific *PANX1* gene expression changes [113]. Our cross-analysis of PANX1 interactors, synaptic-expressed genes, and genetic risk susceptibility to neurological conditions is a key step in bridging our gaps in understanding how PANX1 is linked to these various conditions.

Given the key synaptic findings from our broader analyses, we thought it prudent to refine our approach with the GO synaptic gene set; however, the results were somewhat surprising. Despite the strong representation of well-established and important synaptic genes associated with neurological conditions in the PANX1 set (refer to Table 3, e.g. *Dpysl2*, *Actr3*, *Hip1*, several *Rab*s etc.), when we restricted our disease-associated analysis to “GO synaptic” genes, we only identified the vesicular trafficking regulator NSF as the sole common gene shared by PANX1 and a neurological disease susceptibility gene set (specifically for Parkinson’s disease). Given that studies in Drosophila have shown that expression of a dominant-negative form of *Nsf2* leads to disrupted neuromuscular junction and synaptic structural development [114,115] and that this is linked to actin-cytoskeleton regulation [116], this finding suggests that investigating the NSF-PANX1 interaction could be of key interest in terms of exploring a putative role for PANX1 in synaptic dysfunction in Parkinson’s disease [117]. The connection to Parkinson’s disease is further bolstered by our PANTHER pathway analysis, which identified Parkinson’s disease as one of six pathways exhibiting enrichment within the PANX1 interactome. Importantly, the limited output of the GO synaptic analysis (one gene identified) underscores a key caveat of bioinformatics and GO, in that many key connections may be missed as the outputs are limited by the inputs (a gene may not have met overly restrictive inclusion criteria or certain literature has been missed). Given this limitation, we broadened our approach to compare enriched synapse-specific GO terms (Table 5). In accordance with casting a wide net, we focused on cellular component locations (“cellular component” domain) and biological processes (“biological process” domain), which are relatively broader than the third GO domain, “molecular function”. We identified similar neurological condition-associated cellular components/processes as those identified in our STRING analysis of PANX1 PPIs, such as “vesicle-mediated transport”. These similarities provide potential avenues of insight into the connection between PANX1 and various neurological conditions, as discussed above, such as the link between vesicle-mediated transport genes and Parkinson’s disease risk. These potential links between PANX1, vesicle transport, and Parkinson’s disease may be worth pursuing given the cell-to-cell propagation of alpha-synuclein fibrils, implicated in Parkinson’s disease, involves macropinocytosis [118], a process in which PANX1 has been implicated [119–121]. Further dissection of brain region-specific expression of PANX1 interactors in relevant animal models could also provide important clues.

### Limitations of GWAS and GO analysis can obscure key synaptic PPIs

GWAS exhibits several key caveats. Importantly, GWAS was historically somewhat restricted to European populations, thereby limiting their broader use and application [122]; importantly, some recent progress has been made in expanding GWAS to more groups, especially Asian populations (see Table 7 for details of GWASs used in the current study). Additionally, although GWAS identify genetic differences associated with disease (and beyond), these are not necessarily causal, and where mechanistic links might be present, these can be challenging and complicated to unravel [123]. In terms of the analysis of synaptic representation within suggestive candidate genes for neurological conditions, there are several types of connections that can be missed, as briefly discussed above. For example, given the limitations of gene ontologies [124], not all proteins that could be present at synapses during their lifespan may have their genes annotated to GO “synapse” – for example, *PANX1* itself is not found in GO “synapse”, despite its described localization and functional characterization at the post-synapse. Similarly, a recently created synaptic gene ontology database (https://www.syngoportal.org/) does not contain *PANX1*. Furthermore, identification of a connection does not imply causality; this requires careful and in-depth follow-up of cell biology studies amongst other analyses. Additionally, the analysis is limited to gene level associations with disease and does not account for disorder/disease-associated differences in expression levels, post-translational modifications or protein-targeting pathophysiological mechanisms, like autoantibody production, all of which have been described for CRMP2 (*e.g.,* changes in protein expression levels in several neurological conditions [125], hyperphosphorylation in Alzheimer’s disease [126–128], and auto-antibodies in autism spectrum disorders [129]). Finally, these genetic approaches cannot account for the influence of environmental factors such as inflammation due to injury or infection, which would be expected to have a major impact on PANX1 function and regulation [111,130–132], including neuronal PANX1. For example, the inflammatory mediator TNF-α increases PANX1 expression and surface localization in human umbilical vein endothelial cells, resulting in increased intracellular Ca^2+^ and release of the pro-inflammatory cytokine IL-β1 [133]. If neuronal PANX1 levels are similarly upregulated by inflammatory mediators, this could inhibit morphological plasticity mechanisms like neurite outgrowth [11,12] and contribute to dendritic spine instability [22–24]. Supporting this, recent work suggests that spine and synaptic plasticity deficits in a mouse model of Alzheimer’s disease can be mitigated by blocking PANX1 [26]. In a similar fashion, PANX1 could be involved in impaired cognitive function associated with a wide variety of diseases and conditions in which heightened peripheral inflammation leads to neuroinflammation (reviewed in [134]), often associated with increases in TNF-α, and as such, could be a potential therapeutic target.

**Table 7.** Overview of the specified study populations in the genome-wide association studies (GWASs) extracted from the GWAS catalog for each neurological condition (prior to applying filtering criteria).

Populations	Autism spectrum disorder (*n* = 49)	Schizophrenia (*n* = 212)	Parkinson’s disease (*n* = 69)	Alzheimer’s disease (*n* = 161)
European	40.8%	45.3%	73.9%	59.0%
East Asian	8.2%	19.8%	8.7%	6.2%
South Asian	-	0.9%	1.4%	-
Asian unspecified	2.0%	2.8%	4.3%	-
Greater Middle Eastern (Middle Eastern, North African or Persian)	4.1%	0.5%	-	2.5%
African American or Afro-Caribbean	2.0%	5.7%	-	6.2%
Hispanic or Latin American	-	2.8%	2.9%	2.5%
Native American	-	0.9%	-	0.6%
Sub-Saharan African	-	0.5%	1.4%	-
African unspecified	2.0%	1.4%	-	0.6%
Oceanian	-	0.9%	-	-
Other	2.0%	2.4%	2.9%	0.6%

### Where do we go from here?

To fully appreciate both the complexity and validity of putative pathophysiological mechanisms hinted at by the analyses presented here, additional work in rodent and human iPSC-derived models of neuropsychiatric conditions would be a logical next step. For example, while we have previously validated interactions of PANX1 with ARP3 and CRMP2 in cell lines, investigating their interplay within living neurons and brain will help to shed light on how this and related PPI networks intersect in synapse development in health and disease states. Finally, we currently have limited insight into how PANX1 protein interactions could be involved in or regulate channel function, or how changes in ion and/or metabolite fluxes may impact the interactions or the function of the interacting proteins. Disentangling the impact of interacting proteins on PANX1 properties like channel function (trafficking, post-translational modifications), and *vice versa*, as well as PANX1 channel-independent signaling (*i.e.*, scaffold or other functions) will be critical for fulsome understanding of the role of PANX1 in brain health and disease.

## Supplementary Material

Supplemental Material

## Data Availability

Information about the publicly available data used in this study is outlined throughout the manuscript and supplemental tables. For the proteomics data, RAW files are deposited in the Center for Computational Mass Spectrometry (MassIVE MSV000093036). Please contact Dr. Leigh Anne Swayne (lswayne@uvic.ca) for additional information, if required. The R code is available and can be obtained from Dr. Simona Denise Frederiksen, upon reasonable request.
